# Тransplastomic tobacco plants producing the hydrophilic domain of the sheep pox virus coat protein L1R

**DOI:** 10.18699/VJ20.689

**Published:** 2020-12

**Authors:** D.K. Beisenov, G.E. Stanbekova, B.K. Iskakov

**Affiliations:** M.A. Aitkhozhin Institute of Molecular Biology and Biochemistry, Almaty, Kazakhstan; M.A. Aitkhozhin Institute of Molecular Biology and Biochemistry, Almaty, Kazakhstan; M.A. Aitkhozhin Institute of Molecular Biology and Biochemistry, Almaty, Kazakhstan

**Keywords:** Sheeppox virus, tobacco, Nicotiana tabacum, L1R protein, chloroplasts, transplastomic plants, вирус оспы овец, Sheeppox virus, табак, Nicotiana tabacum, белок L1R, хлоропласты, транспластомные растения

## Abstract

Sheep pox has a wide geographical range of distribution and poses a threat to sheep breeding worldwide, as the disease is highly contagious and is accompanied by large economic losses. Vaccines based on live attenuated virus strains are currently being used for prevention of this disease. Such vaccines are effective, but potentially dangerous because of the possible virus reversion to a pathogenic state. The development of safe recombinant subunit vaccines against sheep pox is very relevant. The high ploidy level of the plant chloroplasts makes it possible to obtain large quantities of foreign proteins. The purpose of this study was to create transplastomic Nicotiana tabacum plants producing one of the candidate vaccine proteins of sheep pox virus L1R. A vector containing a deletion variant of the SPPV_56 gene, which encodes the N-terminal hydrophilic part of the viral coat protein L1R, was constructed to transform tobacco plastids. It provides integration of the transgene into the trnG/trnfM region of the chloroplast tobacco genome by homologous recombination. Spectinomycin-resistant tobacco lines were obtained by biolistic gun-mediated genetic transformation. PCR analysis in the presence of gene-specific primers confirmed integration of the transgene into the plant genome. Subsequent Northern and Western blot analysis showed the gene expression at the transcriptional and translational levels. The recombinant protein yields reached up to 0.9 % of total soluble protein. The transplastomic plants displayed a growth retardation and pale green leaf color compared to the wild type, but they developed normally and produced seeds. Southern blot analysis showed heteroplasmy of the plastids in the obtained plants due to recombination events between native and introduced regulatory plastid DNA elements. The recombinant protein from plant tissue was purified using metal affinity chromatography. Future research will be focused on determining the potential of the chloroplast-produced protein to induce neutralizing antibodies against SPPV strains.

## Abstract (ru)

Оспа овец имеет широкий географический ареал и представляет угрозу овцеводству во всем мире, так как заболевание высококонтагиозное и сопровождается большими экономическими потерями. В настоящее время для профилактики этого заболевания применяются вакцины на основе живых аттенуированных штаммов вируса. Подобные вакцины эффективны, однако потенциально опасны из-за возможной реверсии вируса к патогенному состоянию, поэтому весьма актуально создание безопасных рекомбинантных субъединичных вакцин против оспы овец. Известно, что хлоропласты растений в силу своей полиплоидности могут нарабатывать чужеродные белки в больших количествах. Целью данного исследования было получение транспластомных растений табака Nicotiana tabacum, синтезирующих один из кандидатных вакцинных белков вируса оспы овец L1R. Для проведения генетической трансформации хлоропластов создана конструкция, обеспечивающая интеграцию делеционного варианта гена SPPV_56, кодирующего N-концевую гидрофильную часть оболочечного белка L1R, в межгенную область trnG/trnfM хлоропластного генома табака путем гомологичной рекомбинации. Методом биобаллистики с помощью «генной пушки» получены линии табака, устойчивые к селективному антибиотику спектиномицину. ПЦР-анализ в присутствии ген-специфичных праймеров подтвердил интеграцию целевой вставки в растительный геном. Последующие нозерн- и вестернблот анализы препаратов РНК и белковых экстрактов из полученных растений показали экспрессию целевого гена на транскрипционном и трансляционном уровне. Содержание рекомбинантного белка составило ~0.9 % от общего растворимого белка. Несмотря на задержку роста и более бледную окраску листьев по сравнению с растениями дикого типа, транспластомные растения нормально развивались и завязывали семена. Оценка гомопластидности методом Саузерна выявила гетерогенность пластидных геномов полученных растений, обусловленную генетической рекомбинацией между эндогенными и привнесенными в составе конструкции хлоропластными регуляторными ДНК-последовательностями. Методом металл-аффинной хроматографии была проведена очистка рекомбинантного белка из растительной ткани. В дальнейшем планируется изучить способность продуцируемого хлоропластами белка индуцировать вируснейтрализующие антитела против штаммов вируса оспы овец.

## Introduction

Sheeppox virus (SPPV) belongs to the Capripoxvirus genus, a member of the Poxviridae family (Tulman et al., 2002). The highly contagious sheeppox disease causes significant economic damage to sheep breeding farms, due to high sheep mortality rate, especially among young animals. It also decrease the productivity of meat and wool, and increase the cost of veterinary and sanitary measures. The geographic distribution of sheep pox is very extensive. The disease is endemic in the Middle East, Central and South Asia, China, Central and North Africa. Outbreaks of sheep pox are regularly recorded in the CIS countries, including Russia and Kazakhstan

Currently available live attenuated vaccines based on “NISKHI” strain are widely used for the specific prophylaxis of sheep pox in Russia and the CIS countries (Kurchenko et al., 1991). Live attenuated vaccines are potentially dangerous and theoretically capable of recombining to form virulent strains. As an alternative, recombinant vaccines containing highly immunogenic coat proteins are effective and safer immunizing drugs. Hepatitis B vaccine produced by incorporating the surface antigen of the virus into the genome of yeast cells is an example (McAleer et al., 1984).

Bacterial, yeast, animal, plant and other systems are currently used for the production of target recombinant proteins. The main disadvantage of prokaryotic systems is the absence of post-translational modification of proteins, while yeasts are characterized by excessive glycosylation of proteins, which is different from mammalian cells. Animal systems for the expression of recombinant proteins are extremely expensive and allow to obtain only small amounts of a pure product (Demain, Vaishnav, 2009).

Plant-based recombinant vaccine is an attractive alternative to other systems due to their low cost and absence of human and animal pathogens. Transgenic plants obtained by nuclear transformation usually produce low level of foreign protein (Shchelkunov et al., 2011). Multiplication of transgene copies in order to increase its expression level often leads to posttranscriptional silencing (Finnegan, McElroy, 1994).

Expression of transgenes in plastids has several important advantages over nuclear expression. The content of the recombinant protein in chloroplasts is several orders of magnitude higher compared to nuclear expression and possible to reach more than 70 % of the total soluble protein (TSP) (Oey et al., 2009). Other advantages are the absence of transgene silencing, protection of the target recombinant proteins in plastids from cellular proteases, integration of the transgene into the same intergenic region of plastid DNA, and the possibility of simultaneous expression of several transgenes by combining them into one operon. In contrast to nuclear transformation, transplastomic plants are safer for agrocenoses, since chloroplasts are not contained in pollen and the transfer of transgenes to closely related plant species is unlikely (Clarke, Daniell, 2011).

Works on Dengue viruses, poliomyelitis, the causative agent of tuberculosis, and smallpox vaccine are examples of successful production of antigens in transplastomic plants (Rigano et al., 2009; Daniell et al., 2019; Saba et al., 2019; van Eerde et al., 2019).

We used SPPV_56 gene in this study, as it encodes an ortholog of the well-studied L1R protein of the vaccinia virus, which was used as a live smallpox vaccine in the 20th century. The L1R is a membrane protein of the infectious intracellular mature virion (IMV) and is required for the virus to enter the cell (Bisht et al., 2008). Bacterially synthesized shortened form of the L1R protein induced the production of virus-neutralizing antibodies to SPPV in immunized laboratory animals (Chervyakova et al., 2016) and gave us the reason to consider it as a candidate subunit vaccine.

The aim of this work is to obtain transplastomic tobacco plants expressing a deletion variant of the SPPV_56 gene encoding a protein domain exposed on the outer side of the virion membrane, which we have chosen as a candidate subunit vaccine and will be designated hereinafter as shL1RΔ.

## Research methods

Development of chloroplast transformation plasmids. We have cloned a 567 bp fragment of the SPPV_56 gene (GenBank ID: NP_659632), encoding the N-terminal hydrophilic part of the shL1R protein, into the pET19b/SPPV_56Δ expression vector (Beisenov et al., 2014). Then the deletion variant of the SPPV gene was sequentially transferred into the pICH11599, pHK20 and pNT4 plasmids, kindly provided by prof. H. Warzecha (Germany). The resulting vector was designated pNT4/shL1RΔ. Thermo produced all enzymes used in this work. The target gene was under the transcriptional control of chloroplast elements: the Prrn promoter of the ribosomal operon and the TrbcL terminator of the gene for the large subunit of ribulose bisphosphate carboxylase. The vector includes the aadA marker gene encoding aminoglycoside adenylyltransferase, which confers antibiotic resistance to spectinomycin and streptomycin and allows selection of transformants. The aadA gene is located between the Prrn promoter and the plastid psbA gene terminator. The flanking sequences ensure the integration of the transgene into the trnG/trnf M intergenic region of the chloroplast genome by homologous recombination.

DNA sequencing was performed using a commercial Big Dye® Terminator v. 3.1 kit (Applied Biosystems) according to the manufacturer’s protocol. Gene-specific 56‑for (5′‑gcat catatgggagcagccgctagtat) and 56‑rev (5′-gcatgtcgacttatatata aaattgatatatccgtatcccga) primers were used for reading in both directions. DNA samples were analyzed on an ABI Prism 310 genetic analyzer (Applied Biosystems).

Chloroplast transformation. We used Nicotiana tabacum (cv. Petit Havana) leaves for transformation. Tobacco plants were grown under aseptic conditions in vitro on MS medium (Murashige, Skoog, 1962) containing 3 % sucrose and 0.7 % agar. Biolistic was performed using a PDS-1000/He Biolistic Particle Delivery System (Bio-Rad) gene gun according to a generally accepted protocol (Svab et al., 1990). Leaf explants were placed for regeneration on Petri dishes with MS medium containing 1 μg/ml BAP (6-benzylaminopurine), 0.1 μg/ml NAA (naphthylacetic acid), and 500 μg/ml of the spectinomycin (Sm). The dishes were incubated at 23 °C, with an illumination of 3000 lux and a light regime of 16/8 hours (day/ night). Leaf segments were transferred to Petri dishes with fresh medium every two weeks. The shoots regenerated for several months were cut and rooted on MS medium without hormones and with Sm antibiotic.

DNA isolation. Total DNA were isolated from 100 mg of tobacco leaves using the DNeasy Plant Mini Kit (Qiagen) according to the manufacturer’s protocol

Polymerase chain reaction (PCR) for the detection of the transgene in plants was carried out in the presence of Taq DNA polymerase (Thermo) and a pair of 56-for/56-rev primers. Apair of trnH-for/aadA-rev primers (5′-cacaatccactgccttgatcc; 5′-agaagaagatcgcttggcctc) were used to detect the recombination variant. 50 ng of total plant DNA was used as a template. The reaction was carried out in the following temperature regime: stage 1 – 3 min at 94 °С (1 cycle); stage 2 – 30 sec at 94 °С, 30 sec at 54 °С, 1 min at 72 °С (30 cycles); stage 3 – 5 min at 72 °С (1 cycle).

Western blotting. Protein preparations from plant leaves were isolated using the Trizol reagent (Sigma) according to the manufacturer’s recommendations. Protein concentration was measured relative to known concentrations of bovine serum albumin (BSA) by M. Bradford method (Bradford, 1976).

АКТУАЛЬНЫЕ ТЕХНОЛОГИИ / MAINSTREAM TECHNOLOGIES 907 DNA sequencing was performed using a commercial Big Dye® Terminator v. 3.1 kit (Applied Biosystems) according to the manufacturer’s protocol. Gene-specific 56‑for (5′‑gcat catatgggagcagccgctagtat) and 56‑rev (5′-gcatgtcgacttatatata aaattgatatatccgtatcccga) primers were used for reading in both directions. DNA samples were analyzed on an ABI Prism 310 genetic analyzer (Applied Biosystems). Chloroplast transformation. We used Nicotiana tabacum (cv. Petit Havana) leaves for transformation. Tobacco plants were grown under aseptic conditions in vitro on MS medium (Murashige, Skoog, 1962) containing 3 % sucrose and 0.7 % agar. Biolistic was performed using a PDS-1000/He Biolistic Particle Delivery System (Bio-Rad) gene gun according to a generally accepted protocol (Svab et al., 1990). Leaf explants were placed for regeneration on Petri dishes with MS medium containing 1 μg/ml BAP (6-benzylaminopurine), 0.1 μg/ml NAA (naphthylacetic acid), and 500 μg/ml of the spectinomycin (Sm). The dishes were incubated at 23 °C, with an illumination of 3000 lux and a light regime of 16/8 hours (day/ night). Leaf segments were transferred to Petri dishes with fresh medium every two weeks. The shoots regenerated for several months were cut and rooted on MS medium without hormones and with Sm antibiotic. DNA isolation. Total DNA were isolated from 100 mg of tobacco leaves using the DNeasy Plant Mini Kit (Qiagen) according to the manufacturer’s protocol. Polymerase chain reaction (PCR) for the detection of the transgene in plants was carried out in the presence of Taq DNA polymerase (Thermo) and a pair of 56-for/56-rev primers. Apair of trnH-for/aadA-rev primers (5′-cacaatccactgccttgatcc; 5′-agaagaagatcgcttggcctc) were used to detect the recombination variant. 50 ng of total plant DNA was used as a template. The reaction was carried out in the following temperature regime: stage 1 – 3 min at 94 °С (1 cycle); stage 2 – 30 sec at 94 °С, 30 sec at 54 °С, 1 min at 72 °С (30 cycles); stage 3 – 5 min at 72 °С (1 cycle). Western blotting. Protein preparations from plant leaves were isolated using the Trizol reagent (Sigma) according to the manufacturer’s recommendations. Protein concentration was measured relative to known concentrations of bovine serum albumin (BSA) by M. Bradford method (Bradford, 1976).

We used 15 μg of each sample for the electrophoretic separation of plant proteins in a 12 % SDS-PAA gel according to the generally accepted method (Laemmli, 1970). Proteins were transferred to a PVDF membrane (Bio-Rad) after electrophoresis by semi-dry electroblotting in transfer buffer (102 mM glycine, 25 mM Tris-HCl, 20 % (v/v) ethanol) at a 0.8 mA/cm2 current for 1 hour. The membrane was blocked in 5 % non-fat milk (Sigma) prepared in TBS buffer (10 mM Tris-HCl pH 7.5, 150 mM NaCl) for 1 hour. Polyclonal rabbit antibodies specific to the shL1RΔ protein (kindly provided by the Research Institute for Biological Safety Problems, Kazakhstan) or mouse antibodies to pentahistidine (5 PRIME) diluted in blocking buffer in 1: 4000 ratio were used as primary antibodies to detect the protein. We used anti-mouse or anti-rabbit IgG conjugated with horseradish peroxidase (Santa Crus Biotechnology) and diluted in blocking buffer at 1:4000 ratio as secondary antibodies. Incubation with antibodies was carried out for 1 hour at room temperature. Antibodies were washed off four times for 5 min with TBST buffer (TBS containing 0.05 % Tween-20). Chemiluminescent Peroxidase Substrate-3 (Sigma) reagent was used as a substrate. The membranes were exposed on X-ray film (USA Scientific). Protein content in tobacco lines was determined densitometrically using the Image J 1.42 (NIH) program relative to known concentrations of purified shL1RΔ protein synthesized in bacteria. The size of the recombinant proteins was calculated using the GelAnalyzer 19.1 software (www.gelanalyzer.com).

Southern blotting. Total DNA isolated from transplastomic lines and wild-type plants was treated with EcoO109I restriction enzyme (Thermo), selected as a result of computer analysis of the nucleotide sequence of tobacco chloroplast DNA (GenBank ID: Z00044) using the SnapGene program (www.snapgene.com). The DNA fragments were transferred onto a positively charged nylon membrane (Macherey-Nagel) after electrophoresis in 0.8 % agarose gel. Hybridization was carried out at 42 °C overnight. The probe was a DIG-labeled PCR product obtained during the amplification of wild-type DNA using a pair of chl-dir/chl-rev primers (5′-cgacggaga gggggtccacc; 5′-gaagcccctttaccattctgtat). Probe labeling and detection of bound DNA fragments were performed using PCR-DIG Probe Synthesis Kit (Roche) and DIG Luminescent Detection Kit (Roche). The membranes were exposed on X-ray film (USA Scientific).

Northern blotting. RNA were isolated using the Trizol reagent (Sigma). 5 μg of RNA was separated by electrophoresis in a formaldehyde-containing 1.2 % agarose gel, transfered to a nylon membrane (Macherey-Nagel) and incubated with a probe at 50 °C overnight. A DIG-labeled PCR product obtained with the participation of the pNT4/shL1RΔ plasmid and the 56-for/56-rev primer pair was used as a probe. Probe labeling and detection of hybridization products were performed with the same reagents used in Southern blotting

Isolation of recombinant protein from leaves. 1 g of leaves was ground with a pestle in a mortar with 8 ml of lysis buffer (50 mM NaH2PO4, 0.3 M NaCl, 2 mM imidazole, 1 % Triton X‑100, 15 mM β‑mercaptoethanol, 2 mM PMSF, pH 8.0). The lysate was centrifuged at 10,000 g for 20 min. 1 ml of Ni-NTA agarose suspension (5 PRIME) was added to the supernatant and the mixture was incubated with shaking for 1 hour on ice. Afterwards, the resin was washed twice with 8 ml of wash buffer (50 mM NaH2PO4, 0.3 M NaCl, 20 mM imidazole). The protein was washed out in seven steps using 7 ml of elution buffer (50 mM NaH2PO4, 0.3 M NaCl, 250 mM imidazole, pH 8.0). The fractions were pooled, dialyzed against potassium phosphate buffer (pH 7.0) and concentrated by ultrafiltration through 3,000 MWCO HY columns (Amicon).

## Results

Genetic construct design and transformation of tobacco chloroplasts

**Fig. 1.**
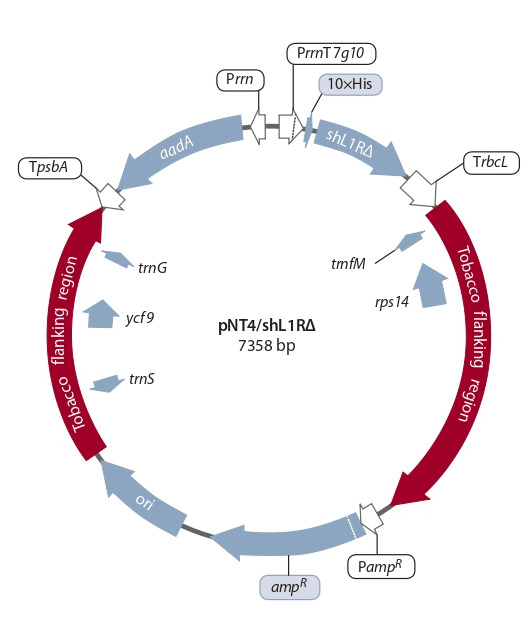
Map of the pNT4/shL1RΔ plasmid. trnG – glycine tRNA gene; TpsbA – terminator of the plastid psbA gene; aadA – spectinomycin resistance gene; Prrn – plastid ribosomal operon promoter; PrrnT7g10 – Prrn fused to the 5’-untranslated region from gene 10 of phage T7; 10×His – a sequence encoding 10 histidine residues; shL1RΔ is a deletion variant of the SPPV_56 gene; TrbcL – terminator of the plastid rbcL gene; trnfM – formyl methionine tRNA gene; ampR and PampR – ampicillin resistance gene and its promoter, respectively; ori – replication start site; ycf9 – gene coding protein Z of photosystem II reaction center; trnS – serine tRNA gene; rps14 – ribosomal protein S14 gene.

We cloned the deletion variant of the SPPV_56 gene (includes the first 567 bp out of 738 bp) into the pNT4 chloroplast vector in three steps to obtain a vector intended for the transformation of chloroplasts. At the first stage, the SPPV gene from the pET19b/SPPV_56Δ plasmid was transferred using NcoI/ BamHI restriction sites into the pICH11599 vector for transient expression, that resulted in pICH11599/SPPV_56Δ plasmid construction. The presence of the XbaI site in the resulting plasmid made it possible to carry out the subsequent cloning of the SPPV gene digested with NcoI/XbaI restriction endonucleases into the intermediate pHK20 vector at the NdeI/ XbaI sites. The insert and the vector were treated with NcoI and NdeI restriction enzymes. Protruding 5′-ends of the DNA were completed with the Klenow fragment in the presence of dNTPs. At the third stage, the SPPV_56Δ gene, the Prrn chloroplast promoter and the TrbcL terminator were transferred at the SacI/HindIII restriction sites into the pNT4 chloroplast vector. Figure 1 shows a schematic representation of the pNT4/ shL1RΔ vector, intended for chloroplasts transformation. Nucleotide sequencing showed absence of any mutations in the SPPV_56Δ gene after cloning and the resulting construct can be used in further work.

**Fig. 2.**
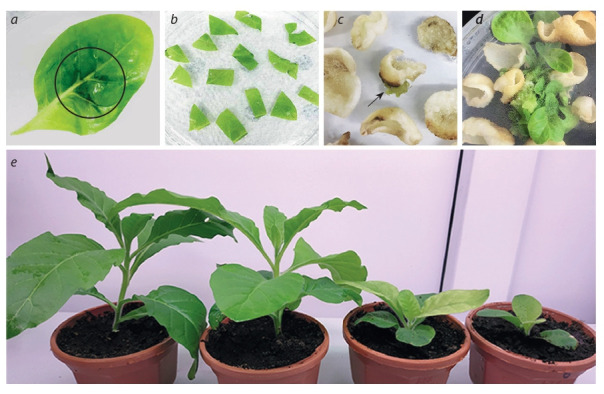
Stages of creating transplastomic tobacco plants. а – a leaf immediately after bombardment (microparticles penetration area is marked); b – leaf segments on the medium for regeneration; c – callus formation (marked by arrow); d – regeneration; e – plants planted in the soil (two wild-type plants on the left, two transplastomic lines on the right).

The process of obtaining transplastomic plants by the biolistic method consisted of several stages: bombardment of whole tobacco leaves with the pNT4/shL1RΔ plasmid immobilized on the surface of gold particles, cultivating them on a medium with hormones and Sm, obtaining calli, selecting regenerating shoots that are resistant to Sm. Figure 2 shows the stages of obtaining transplastomic tobacco plants. As a result, three Sm-resistant shoots were selected from separate segments of eight shot leaves.

Molecular and genetic analysis of the resulting plants

**Fig. 3.**
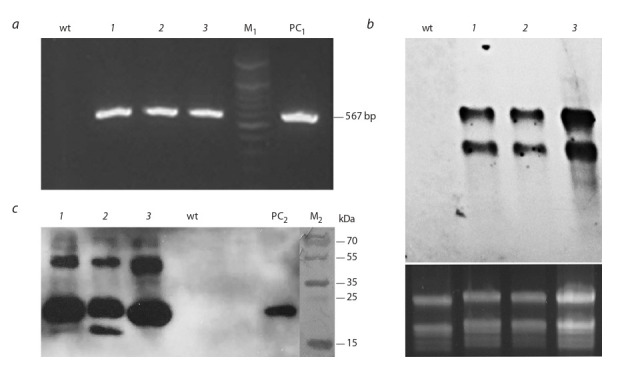
Molecular and genetic analysis of transplastomic plants a – PCR analysis of total DNA from various lines with SPPV_56Δ gene specific primers; b – Northern blotting of total RNA with SPPV_56Δ probe (lower gel stained with ethidium bromide reflects the amount of analyzed RNA); c – Western blotting of protein extracts with antibodies against shL1RΔ. wt – negative control (DNA from wild-type tobacco plant); 1–3 – analyzed plant lines, M1 – DNA marker Gene Ruler 100 bp (Thermo); PC1 – 10 ng pNT4/shL1RΔ; M2 – protein marker PageRuler Plus (Thermo); PC2 – 20 ng of shL1RΔ protein purified from bacteria.

Plants were screened for the presence of the target gene using the PCR method. Total DNA preparations were isolated from the leaves of the tested plants and wild-type plant, which were analyzed using a pair of gene-specific 56‑for/56‑rev primers. The test showed that all three selected lines contained the SPPV_56Δ gene of the expected size (567 bp) (Fig. 3, a). The ability of the resulting lines to express the target gene was studied at the transcriptional and translational levels. Northern blotting of total cellular RNA preparations using a DIGlabeled probe to the SPPV_56Δ gene revealed the presence of two types of recombinant mRNA in all obtained lines (see Fig. 3, b). Along with the monocistronic transcript, a longer product was found, apparently due to ineffective transcription termination, which is generally typical for plastids (Zhou et al., 2007; Oey et al., 2009).

The ability of transplastomic lines to produce the recombinant shL1RΔ protein was assessed by immunoblotting using antibodies to bacterially synthesized shL1RΔ. The recombinant protein in plant extracts corresponds to the theoretically expected size of 23 kDa (see Fig. 3, c). We also identified a 46 kDa protein, which, presumably, is a dimeric form of shL1RΔ. Comparative densitometric analysis of protein bands relative to known amounts of purified bacterially synthesized shL1RΔ in three independent experiments showed that the level of recombinant protein in plants reaches ~0.9 % of the total soluble protein.

Assessment of the homoplasticity of transplastomic plants To obtain homoplastic plants, each line was subjected to further selection in order to eliminate wild-type and select transformed plastids. Leaf segments of the lines were cultivated on a medium with hormones and antibiotic until the appearance of secondary regenerants. This procedure was carried out four times. Then the plants of the T0 generation were planted in the ground for further analysis. Transplastomic lines transplanted into soil, showed signs of growth retardation and paler leaf color in comparison with the wild type plants (see Fig. 2, e). Despite this circumstance, all lines were fertile and formed viable seeds upon self-pollination.

**Fig. 4.**
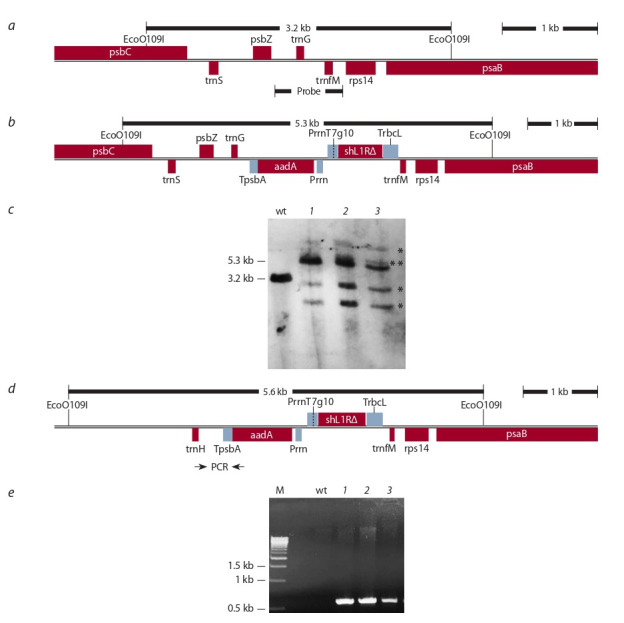
Homoplasmy assessment of transplastomic plants а – the region of transgene integration into the plastid genome of wild-type plants; b – the same region in the transplastomic lines; c – Southern blot analysis with probe to the SPPV_56Δ gene; d – a recombination variant; e – PCR analysis of lines with a pair of trnH-for/aadA-rev primers. wt – a wild-type plant; M – DNA marker Gene Ruler 1 kb (Thermo); 1–3 – analyzed lines.

The homoplasticity of the obtained lines was assessed by the restriction fragment length polymorphism using Southern blotting with labeled probe that covered the site of transgene insertion into plastid DNA between the trnG and trnf M genes with adjacent regions (Fig. 4, a). Analysis showed, the probe bound to one 3.2 kb DNA fragment of the expected length from wild-type plants (see Fig. 4, c). In transplastomic lines, in addition to the expected 5.3 kb fragment we revealed several additional fragments (marked in Fig. 4, c with asterisks). Possibly, plastids heterogeneity revealed in lines is caused by intermolecular post-transformation recombination between endogenous and plastid regulatory elements introduced into the structure: promoters, terminators, 5′-untranslated sequences. Such cases are described in a number of works (McCabe et al., 2008; Zhou et al., 2008; Gray et al., 2009). One of the recombination variants identified in this work (marked in Fig. 4, c with two asterisks and schematically shown in Fig. 4, d ) serves as evidence of the rearrangements that have taken place. The fact of recombination between the natural and introduced plastid TpsbA terminators was confirmed by the presence of the 594 bp DNA fragment of the expected size amplified during PCR analysis with the trnH-for/aadArev primers (see Fig. 4, e). We did not study the rest of the recombination variants.

**Fig. 5.**
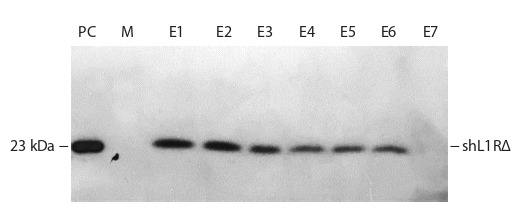
Western blot analysis of fractions eluted from a Ni2+-NTA agarose. PC – 40 ng of shL1RΔ protein produced in bacteria; M – protein marker PageRuler Plus (Thermo); E1– E7 – eluted fractions.

Purification of recombinant protein from plant material The presence of decahistidine at the N-terminus of the recombinant shL1RΔ protein facilitates its further purification by metal affinity chromatography on Ni-NTA agarose. Preliminary experiments showed that the addition of the non-ionic detergent Triton X-100 to the extraction buffer provided a higher yield of the target protein as compared to Tween-20 and SDS. Figure 5 shows the results of immunodetection of the shL1RΔ protein in purified fractions. Protein-containing fractions were pooled, then dialyzed against potassium phosphate buffer and concentrated by ultrafiltration. The yield of the recombinant protein purified from the leaves was 10.3 μg/g

## Discussion

The development of genetic engineering and biotechnology over the past decades has opened up wide opportunities for obtaining a new generation of vaccines based on highly immunogenic surface antigens of human and animal pathogens. The plastid and the transient plant expression systems are cheap source of recombinant proteins for medical and veterinary purposes

In this work, we described the production of transplastomic plants producing one of the candidate vaccine proteins, namely the truncated form of the structural L1R protein of the sheeppox virus. Earlier, we obtained transgenic rapeseed plants with the nuclear localization of the same gene (Beisenov et al., 2019). The content of recombinant viral protein in rape plants was about 0.1 % of the TSP. In this study, we managed to significantly increase the expression of the target gene by transferring it to the chloroplast genome. The content of the recombinant protein was about 0.9 % of the TSP. Potentially, the content of the recombinant protein may be increased by inserting an artificially synthesized gene with a codon optimized for expression in chloroplasts, as demonstrated for antigens of the human papillomavirus (Lenzi et al., 2008; Daniell et al., 2019).

Alternative approaches to increasing the protein content are to increase the copy number of the gene by integration into the inverted repeat region of the plastid genome, and the addition of certain N-terminal peptides in the case of unstable recombinant proteins (Bock, 2014). The problem of heterogeneity of the plants obtained in this work can be solved by reorganizing the genetic structure intended for transformation. F. Zhou et al. changed the orientation of the target gene relative to the aadA gene. As a result, increased distance between the two Prrn promoters made possible to obtain stable homoplastic plants producing antigens of the human immunodeficiency virus at a 40 % of the TSP (Zhou et al., 2008). Nevertheless, the achieved level of synthesis allows us to isolate a sufficient amount of protein required for further immunological studies. We intend to study the ability of the recombinant viral protein shL1RΔ purified from plants to induce the production of virus neutralizing antibodies in laboratory animals.

## Conclusion

As a result of our studies, we have shown the ability to synthesize the shortened structural protein shL1R of sheeppox virus in transplastomic tobacco plants. The recombinant protein can then be used to develop a subunit vaccine against sheep pox.

## Conflict of interest

The authors declare no conflict of interest.
